# The immunocytokine NHS-IL12 as a potential cancer therapeutic

**DOI:** 10.18632/oncotarget.1853

**Published:** 2014-03-24

**Authors:** Jonathan Fallon, Robert Tighe, Giorgio Kradjian, Wilson Guzman, Anna Bernhardt, Berend Neuteboom, Yan Lan, Helen Sabzevari, Jeffrey Schlom, John W. Greiner

**Affiliations:** ^1^ Laboratory of Tumor Immunology and Biology, Center for Cancer Research, National Cancer Institute, Bethesda, Maryland USA;; ^2^ EMD Serono Research and Development Institute, Billerica, Massachusetts USA

**Keywords:** immunocytokine, interleukin-12, tumor necrosis therapy, T cells, immunotherapy

## Abstract

Targeted delivery of IL-12 might turn this cytokine into a safer, more effective cancer therapeutic. Here we describe a novel immunocytokine, NHS-IL12, consisting of two molecules of IL-12 fused to a tumor necrosis-targeting human IgG1 (NHS76). The addition of the human IgG1 moiety resulted in a longer plasma half-life of NHS-IL12 than recombinant IL-12, and a selective targeting to murine tumors *in vivo*. Data from both in vitro assays using human PBMCs and *in vivo* primate studies showed that IFN-gamma production by immune cells is attenuated following treatment with the immunocytokine, suggesting an improved toxicity profile than seen with recombinant IL-12 alone. NHS-IL12 was superior to recombinant IL-12 when evaluated as an anti-tumor agent in three murine tumor models. Mechanistic studies utilizing immune cell subset-depleting antibodies, flow cytometric methods, and *in vitro* cytotoxicity and ELISA assays all indicated that the anti-tumor effects of NHS-IL12 were primarily CD8+ T cell-dependent and likely IL-12-mediated. Combining NHS-IL12 treatment with a cancer vaccine, radiation, or chemotherapy resulted in greater anti-tumor effects than each individual therapy alone. These preclinical findings provide a rationale for the clinical testing of this immunocytokine, both as a single agent and in combination with vaccines, radiation and chemotherapy.

## INTRODUCTION

Interleukin-12 (IL-12) is a pleiotropic proinflammatory cytokine produced by activated dendritic cells (DCs) that is a key mediator of the transition from innate to adaptive immunity [1]. IL-12 promotes cell-mediated immunity by inducing the differentiation of T_H_1 cells [2] and by increasing the proliferation [3, 4] and lytic capacity [5-7] of cytotoxic T and NK cells. High levels of IFN-gamma are produced by T cells and NK cells in response to IL-12 [8], leading to enhanced antigen-presentation through paracrine upregulation of MHC class I and class II expression [9, 10]. In addition, the IL-12–induced release of IFN-gamma stimulates macrophages to produce the anti-angiogenic chemokines CXCL10/IP-10 (interferon-inducible protein 10) and CXCL9/Mig (monokine induced by interferon gamma) [11, 12]. By virtue of its potent abilities to stimulate cell-mediated immunity and inhibit angiogenesis, exogenous IL-12 administration has long been considered a promising anti-cancer treatment [13]. Early preclinical studies of recombinant murine (mu) IL-12 reported dramatic, even curative efficacy against syngeneic murine tumors of various types [14, 15], paving the way for subsequent clinical studies in human subjects. However, after over a decade in early clinical development, documented cases of severe toxicity [16] and generally low response rates [17] to rIL-12 have stymied its clinical development. To successfully exploit the therapeutic potential of IL-12 in the clinic, innovative approaches for improving its tolerability are clearly required.

One strategy for improving the safety of proinflammatory cytokines, such as IL-2 and IL-12, is to direct their delivery to tumors via fusion to a tumor-targeting antibody. Such antibody-cytokine fusion proteins, or “immunocytokines,” have previously demonstrated the ability to enhance anti-tumor immunity in preclinical models [18]. To maximize immunocytokine tolerability, the antibody selected as a vehicle must bind specifically to an antigen uniquely found in tumors. Antibodies directed against necrosis-associated antigens, which are abundantly present in tumors but not in normal tissues, offer an attractive delivery approach [19, 20]. The ability of DNA/histone-binding antibodies to selectively target to regions of tumor necrosis has been well studied both preclinically and clinically [21]. Taking advantage of this concept, a necrosis-targeted IL-12 immunocytokine, called NHS-IL12, was engineered by genetically fusing 2 human IL-12 heterodimers to the C-termini of the heavy chains of the NHS76 antibody. NHS76 is a fully human, phage display-derived IgG1 antibody selected for its specific ability to bind to necrotic regions and thereby target to tumors *in vivo* [22].

Here we describe the *in vivo* pharmacokinetic and tumor-targeting properties, anti-tumor activity, and immunological mechanisms of action of NHS-IL12. For murine studies, a surrogate immunocytokine, NHS-muIL12, was engineered which consists of the human NHS76 antibody fused to 2 murine IL-12 heterodimers. The use of a surrogate molecule was necessary because human IL-12 lacks biological activity in the mouse [23]. To study the full mechanistic potential of the molecule, several syngeneic tumor models were used in immunocompetent mice. The efficacy and immunomodulatory activity of NHS-muIL12 were compared against administrations of non-targeted recombinant muIL-12 (rMuIL-12). In addition, the anti-tumor effects of NHS-muIL12 were studied in combination with radiation, three approved chemotherapeutic agents, and an experimental cancer vaccine.

## RESULTS

### Comparative stimulation of IFN-gamma production by NHS-huIL12 and recombinant human IL-12 (rHuIL-12) *in vitro*

**Figure 1:**
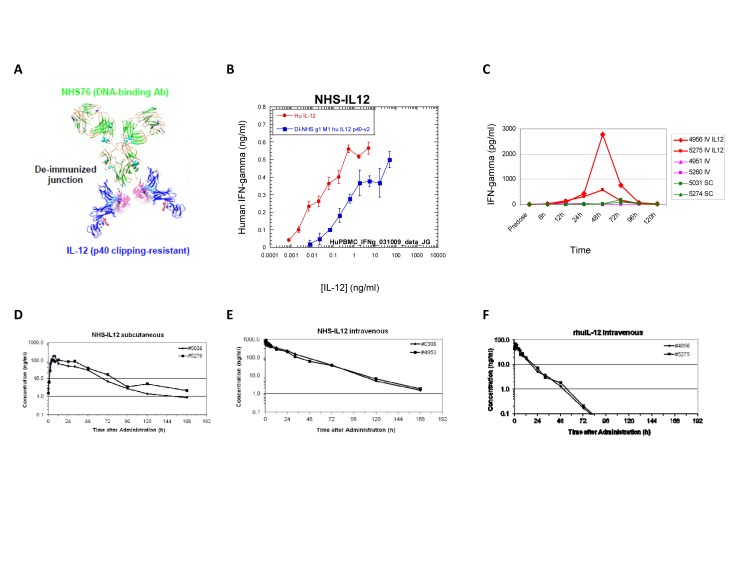
Conjugation of human IL-12 to a DNA/histone-binding antibody increases its half-life and attenuates its ability to stimulate IFN-gamma production (A) Ribbon diagram of NHS-IL12, an immunocytokine consisting of the NHS76 antibody fused to 2 IL-12p70 heterodimers. (B) Human PBMCs were stimulated *in vitro* with 2 μg/ml PHA for 4 days, and then an additional day following the addition of 10ng/ml rHuIL-2. NHS-huIL12 (blue) or rHuIL-12 (red) was then added to the cells, and supernatant samples were collected 24 h later. Human IFN-gamma levels in the supernatant were determined by ELISA. Results show mean ± SE of triplicate wells for each condition. (C) Cynomolgus macaques were treated with 40 μg/kg NHS-huIL12 s.c. (green), 40 μg/kg NHS-huIL12 intravenously (i.v., pink), or 4 μg/kg rHuIL-12 i.v. (red). Serum IFN-gamma levels in 2 animals per group were monitored for 5 days after injection. (D-F) Pharmacokinetic profile of NHS-huIL12 compared to rHuIL-12 in cynomolgus macaques. Cynomolgus macaques were treated with (D) 40 μg/kg NHS-huIL12 s.c., (E) 40 μg/kg NHS-huIL12 intravenously (i.v.), or (F) 4 μg/kg rHuIL-12 i.v. Plasma drug levels in 2 animals per treatment group were monitored for 8 days following injection.

Figure 1A shows a ribbon diagram that illustrates the immunocytokine NHS-IL12, which consists of the tumor necrosis-targeting NHS76 antibody fused to 2 molecules of the human IL-12 heterodimer. *In vitro* studies using PHA-stimulated human PBMCs (Figure 1B) or human NK-92 cells (data not shown) revealed that IFN-gamma production was severely attenuated following the addition of the immunocytokine. Since IFN-gamma production has been linked with rHuIL-12 toxicity [16], these findings suggest that delivering IL-12 via the immunocytokine might result in a better tolerated and safer therapeutic.

### Pharmacokinetics and in vivo bioactivity of NHS-huIL12 treatment in cynomolgus monkeys

In subsequent *in vivo* studies, single s.c. or i.v. injections of human NHS-huIL12 (40 μg/kg) or rHuIL-12 (4 μg/kg) were administered to cynomolgus macaques (*n* = 2/cohort) to examine both IFN-gamma serum levels as well as comparative pharmacokinetics. Consistent with the previous *in vitro* assays, administration of NHS-huIL12 induced much lower serum IFN-gamma levels than did rHuIL-12 (Figure 1C). Pharmacokinetic analyses clearly showed the sustained presence of plasma NHS-huIL12 levels when injected by either route (Figure 1D-E), as compared with a single i.v. injection of 4 μg/kg rHuIL-12 (Figure 1F). NHS-huIL12 was well tolerated as evidenced by (a) an absence of any measurable increase in body temperature, (b) a minor reduction in platelet counts, and (c) no significant injection site reactions. Immunogenicity against the fully humanized NHS-huIL12 was observed in these monkeys, with a preponderance of reactivity against the Ig, rather than the IL-12 portion (data not shown).

### *In vivo* tumor-targeting and efficacy of NHS-muIL12

**Figure 2:**
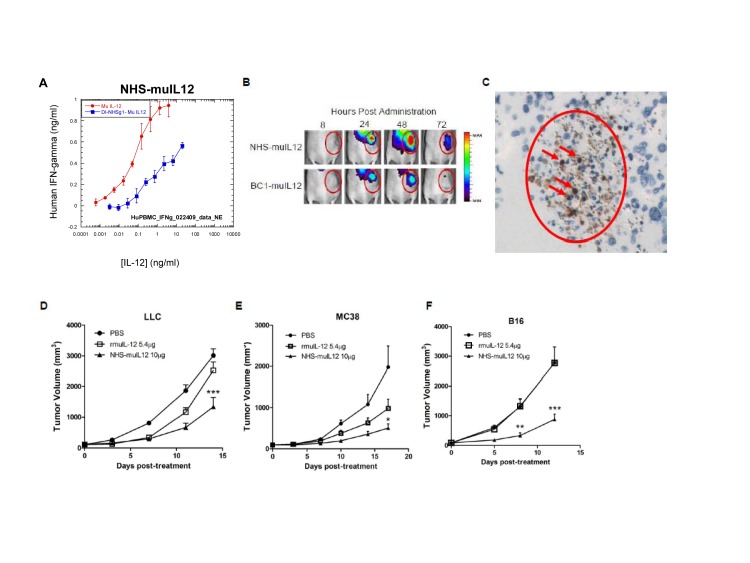
Fusion of murine IL-12 to NHS76 promotes its uptake by tumors and improves its anti-tumor efficacy (A) Human PBMCs were stimulated *in vitro* with 2 μg/ml PHA for 4 days and then an additional day in the presence of 10ng/ml rHuIL-2. NHS-muIL12 (blue) or rMuIL-12 (red) were then added to the cells, and supernatant samples were collected 24 h later. Human IFN-gamma levels in the supernatant were determined by ELISA. Results show mean ± SE of triplicate wells for each condition. (B) Athymic mice bearing subcutaneous LLC tumors were injected with 100 μg of fluorescence-conjugated NHS-muIL12 or BC1-muIL12 and imaged at 8, 24, 48, and 72 h post-injection. One representative mouse from each treatment group is shown. (C) LLC tumor sections revealed that NHS-muIL12 binds to exposed cell nuclei within necrotic regions of murine tumors. (D-F) NHS-muIL12 displayed greater anti-tumor activity than an equimolar dose of rMuIL-12 against subcutaneous (D) LLC, (E) MC38, and (F) B16 tumors (*n* = 8 mice per group). Average tumor volumes ± SE are shown. Asterisks indicate statistically significant differences between the NHS-muIL12 treatment group versus the control or rMuIL-12 treatment groups (2-way ANOVA followed by Bonferroni's post-test; *, *P* < 0.05; **, *P* < 0.01; ***, *P* <0.001).

In order to assess the tumor-targeting and anti-tumor efficacy of NHS-IL12, a second molecule was constructed that included the same human NHS76 antibody, but fused with 2 murine IL-12 heterodimers (NHS-muIL12). That immunocytokine also induced lower levels of IFN-gamma than rMuIL-12 when added to human PBMCs *in vitro* (Figure 2A). *In vivo* fluorescence imaging was used to study the tumor-targeting characteristics of subcutaneously (s.c.) administered NHS-muIL12 in mice bearing s.c. LLC tumors. Localization of fluorescent-labeled NHS-muIL12 within tumors was compared against BC1-muIL12, a human monoclonal antibody immunocytokine analogous in structure to NHS-muIL12 but lacking a target antigen in murine tumors [24, 25]. Following subcutaneous delivery at a distal site, NHS-muIL12 accumulated within tumors to a higher level than did BC1-muIL12 (Figure 2B). Quantitation of tumor accumulation of both immunocytokines is shown in Supplemental Figure S1A, also indicating some passive targeting mechanism of the BC1-muIL12.

To visualize the subcellular localization of NHS-muIL12 *in vivo*, tumor-bearing mice were treated with the molecule, the tumors were harvested and fixed, and immunohistochemical detection was performed against the NHS76 antibody (human IgG1). Strong, distinct staining in the nuclei of tumor cells was observed (Figure 2C), in agreement with the proposed DNA-binding mechanism of the molecule. NHS-muIL12 is a large macromolecular protein that cannot pass through intact cell membranes; therefore, the intracellular penetration of the molecule *in vivo* suggests a loss of cell membrane integrity (i.e., a necrotic/apoptotic phenotype).

In comparative studies, NHS-muIL12 was administered via the s.c. route, as this resulted in elevated levels of tumor uptake but reduced levels of liver uptake than the i.v. route (Supplemental Figure S1B). NHS-muIL12 (10μg, corresponding to a dose of 5.4μg of murine IL-12 equivalents) demonstrated greater anti-tumor effects compared with rMuIL-12 (5.4μg) in 3 different syngeneic tumor models (Lewis lung carcinoma (LLC); MC38 colon carcinoma; B16 melanoma) (2-way ANOVA with Bonferroni post-test, *P* < 0.050) (Figure 2D–F). Both molecules were tolerated equally well as indicated by normal body weight gain (data not shown).

### Dose-dependent anti-tumor activity of NHS-muIL12

**Figure 3:**
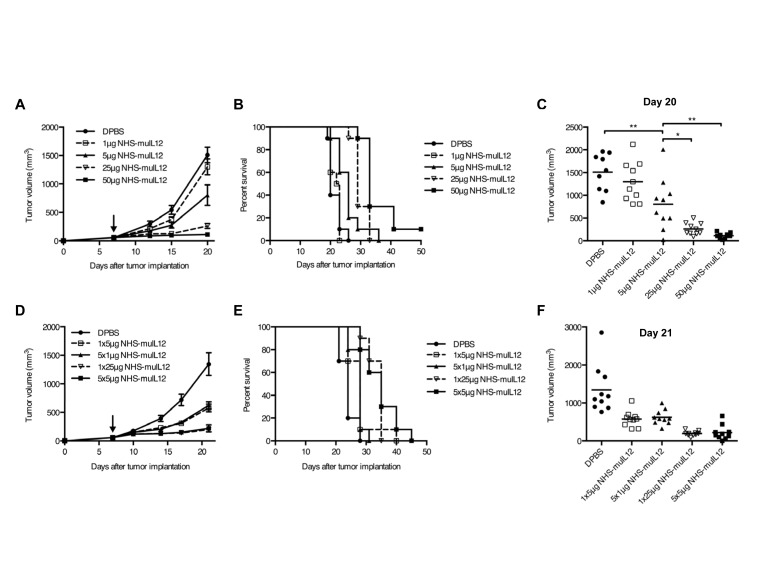
The anti-tumor activity of NHS-muIL12 is dose-dependent and is unaffected by dose fractionation (A–C) Immunocompetent C57BL/6 mice bearing subcutaneous MC38 tumors were randomized (*n* = 9-10 mice per group) and treated with a single s.c. injection of DPBS (closed circle) or 1 μg (open square), 5 μg (closed triangle), 25 μg (open triangle), or 50 μg (closed square) NHS-muIL12. Graphs of (A) mean tumor volume, (B) overall survival, and (C) individual tumor volumes on day 20 are shown. Arrow in panel A indicates treatment on day 7, and error bars indicate SEM. Asterisks indicate statistically significant differences between mean tumor volumes on day 20 (1-way ANOVA with Tukey's post-test; *, *P* < 0.05; **, *P* < 0.01). (D–F) To determine whether dose fractionation affected the anti-tumor activity of NHS-muIL12, MC38 tumor-bearing mice were randomized (*n* = 10 mice per group) and treated with DPBS (closed circle) or 1x5 μg (open square), 5x1 μg (closed triangle), 1x25 μg (open triangle), or 5x5 μg (closed square) NHS-muIL12. Graphs of (D) mean tumor volume, (E) overall survival, and (F) individual tumor volumes on day 21 are shown. Arrow in panel D indicates treatment initiation on day 7, and error bars indicate SEM. A 1-way ANOVA followed by Tukey's multiple comparisons test were used to compare mean tumor volumes on day 21. The mean differences observed at this time point between each NHS-muIL12 treatment group relative to the control group were statistically significant (*P* < 0.0001).

To study the anti-tumor response to NHS-muIL12 over a range of doses, C57BL/6 mice bearing s.c. MC38 tumors were treated with DPBS or a single subcutaneous injection of 1 μg, 5 μg, 25 μg, or 50 μg NHS-muIL12. The delay in tumor outgrowth (Figure 3A), improvement in overall survival (Figure 3B), and reduction in tumor burden (Figure 3C) were all dose-dependent. Differences in overall survival between treatment groups were statistically significant (log-rank test, *P* < 0.0001), and tumor volumes on day 20 correlated with the dose of NHS-muIL12 (linear trend test, *P* < 0.0001).

### Fractionated dosing of NHS-muIL12

With knowledge that the human NHS76 antibody would elicit a xenogeneic immune response over time (Supplemental Figure S2A-B), multiple daily dosing regimens were evaluated. Mice bearing s.c. MC38 tumors were injected subcutaneously with DPBS or NHS-muIL12 according to 1 of the following schedules: a single injection of 5 μg or 25 μg NHS-muIL12, or 5 daily injections of 1 μg or 5 μg each. In this study, the total dose of NHS-muIL12 given determined the delay in tumor outgrowth (Figure 3D), overall survival (Figure 3E), and tumor burden (Figure 3F). However, fractionated dosing did not appear to have a major advantage over single dosing of NHS-muIL12 at either total dose level (*P* > 0.05).

### Immune correlates of NHS-muIL12 function

**Figure 4:**
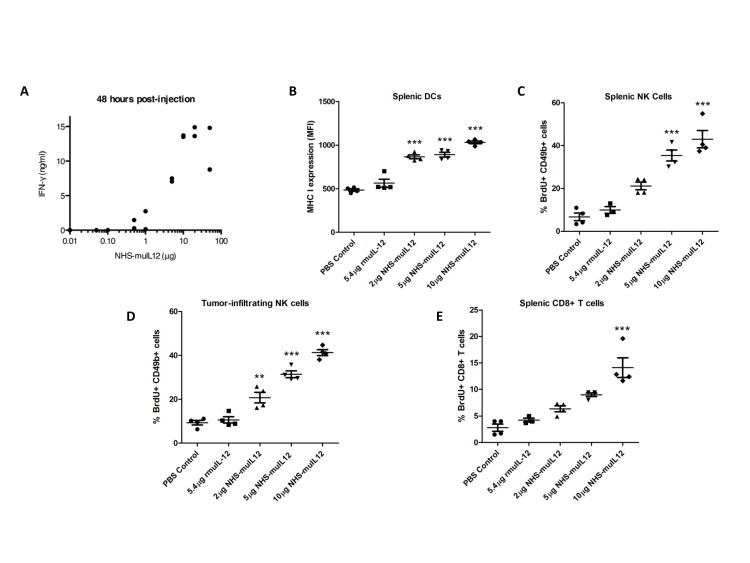
Immune activation markers correlate with increasing doses of NHS-muIL12 (A) C57BL/6 mice were injected with a single dose of NHS-muIL12 within the range of 0.01–50 μg. For each dose level, 2 mice were injected and serum samples were obtained 48 h later for IFN-gamma detection by ELISA. (B–E) MC38 tumor-bearing mice were randomized (*n* = 4 mice per group) and treated with PBS (closed circle), 5.4 μg rMuIL-12 (closed square), 2 μg NHS-muIL12 (closed triangle), 5 μg NHS-muIL12 (closed inverted triangle), or 10 μg NHS-muIL12 (closed diamond). (B) Flow cytometry was used to quantify MHC class I expression levels on splenic dendritic cells 5 days after treatment. Percent BrdU incorporation by (C) splenic NK, (D) tumor-infiltrating NK, and (E) splenic CD8+ T cells was also evaluated 5 days after treatment by flow cytometry. Error bars indicate SEM. Asterisks indicate statistically significant differences between NHS-muIL12 treated groups versus control and rMuIL-12 treated groups (1-way ANOVA with Bonferroni's post-test; **, *P* < 0.01; ***, *P* <0.001).

NHS-muIL12 did not slow the rate of MC38 tumor cell growth directly *in vitro* (data not shown), suggesting that an immune mechanism(s) was responsible for the anti-tumor effects seen *in vivo*. IL-12 is known to induce the release of IFN-gamma, so it was not surprising that NHS-muIL12 induced a dose-dependent serum IFN-gamma response *in vivo* (Figure 4A). In addition, NHS-muIL12 induced the *in vivo* maturation of splenic dendritic cells, as determined by quantifying MHC class I expression on DCs (Figure 4B). NHS-muIL12 also induced the *in vivo* activation of splenic NK cells (Figure 4C), tumor-infiltrating NK cells (Figure 4D), and splenic CD8+ T cells (Figure 4E) in MC38 tumor-bearing mice. Similar results were seen in B16 tumor-bearing mice treated with NHS-muIL12 (Supplemental Figure S3A–D). All of these effects were dose-dependent, and NHS-muIL12 was more effective at activating the immune response than an equimolar dose of rMuIL-12 for all of these parameters (Figure 4B–E and Supplemental Figure S3A–E). These correlates of immune response and anti-tumor efficacy might prove useful to monitor in NHS-IL12‒treated patients.

### CD8+ T-cell activation by NHS-muIL12

**Figure 5:**
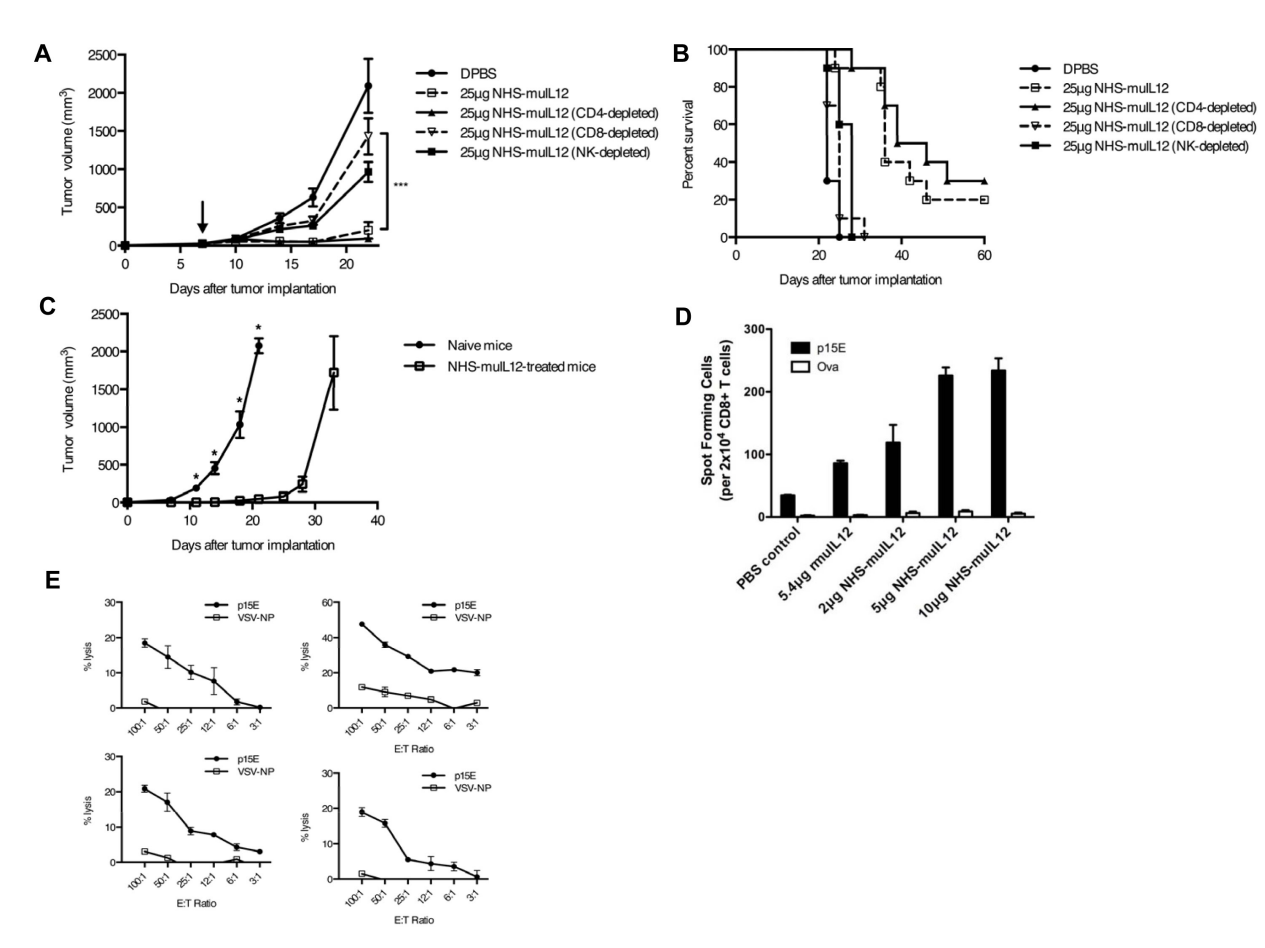
The anti-tumor activity of NHS-muIL12 depends on CD8+ T cells (A–C) MC38 tumor-bearing mice were randomized and treated with DPBS (closed circle), 25 μg NHS-muIL12 (open square), or 25 μg NHS-muIL12 together with antibodies to deplete CD4 (closed triangle), CD8 (open triangle), or NK cells (closed square). Arrow denotes NHS-muIL12 treatment on day 7. Graphs representing (A) mean tumor volume over time and (B) overall survival are shown. Differences in mean tumor volumes on day 22 between mice treated with 25 μg NHS-muIL12 alone and together with CD8+ cell-depleting antibodies were statistically significant (1-way ANOVA with Bonferroni's post-test, ***, *P* < 0.001). (C) Four mice from this study became tumor-free following NHS-muIL12 treatment, and were rechallenged 4 months later with 300,000 MC38 cells on the opposite rear flank (open square). Five naïve C57BL/6 mice were challenged with MC38 tumors in the same way (closed circle) (*t*-test; *, *P* < 0.05). Error bars in panels A and C indicate SEM. (D) MC38 tumor-bearing mice were randomized and treated with PBS, 5.4 μg rMuIL-12, 2 μg NHS-muIL12, 5 μg NHS-muIL12, or 10 μg NHS-muIL12. Five days after treatment, splenocytes from 4 mice per treatment group were pooled and stimulated for 6 days *in vitro* with 1 μg/ml p15E peptide. ELISPOT assays were then used to determine the relative frequencies of splenic CD8+ T cells specific for p15E or ovalbumin. (E) The spleens of 4 CEA.Tg mice that were completely cured of MC38/CEA+ tumors following NHS-muIL12 treatment were tested individually for the presence of a p15E-specific CTL response. Results shown in panels D and E are mean ± SE of triplicate wells.

To identify the effector cells responsible for the anti-tumor activity of NHS-muIL12, MC38 tumor-bearing mice were treated with NHS-muIL12 in the presence or absence of immune cell subset-depleting antibodies. The *in vivo* depletion of CD8+ T cells, but not CD4+ cells, resulted in a substantial loss of the antitumor efficacy following the administration of NHS-muIL12 (Figure 5A–B). Depletion of NK cells also appeared to abrogate the antitumor efficacy of NHS-muIL12, but this effect was not statistically significant. Four mice treated with NHS-muIL12 in this study had a durable tumor regression. When rechallenged with MC38 tumor cells on the opposite rear flank, tumor outgrowth was significantly delayed compared to naïve controls (Figure 5C), indicating that the previously treated mice had developed a systemic anti-tumor memory response.

Although CD8+ T cells were shown to contribute to the anti-tumor effects of NHS-muIL12, the antigen specificity of this CD8+ T-cell response was unclear. The p15E endogenous retroviral antigen has previously been shown to be a tumor rejection antigen for several murine tumor models including MC38 [26], suggesting that this antigen may also be important in this system. ELISPOT assays detected a high frequency of p15E-specific T cells in NHS-muIL12–treated mice bearing MC38 tumors (Figure 5D), and similar results were observed in the B16 tumor model (Supplemental Figure S3E). In a separate experiment, mice treated with NHS-muIL12 and cured of their tumors were found to have a detectable p15E-specific CD8+ lytic T-cell response (Figure 5E). These immune-mediated anti-tumor effects suggest that NHS-muIL12 might further enhance the activity of cancer vaccines or immune-modulating agents.

### Combination therapy with NHS-muIL12 and vaccine

**Figure 6:**
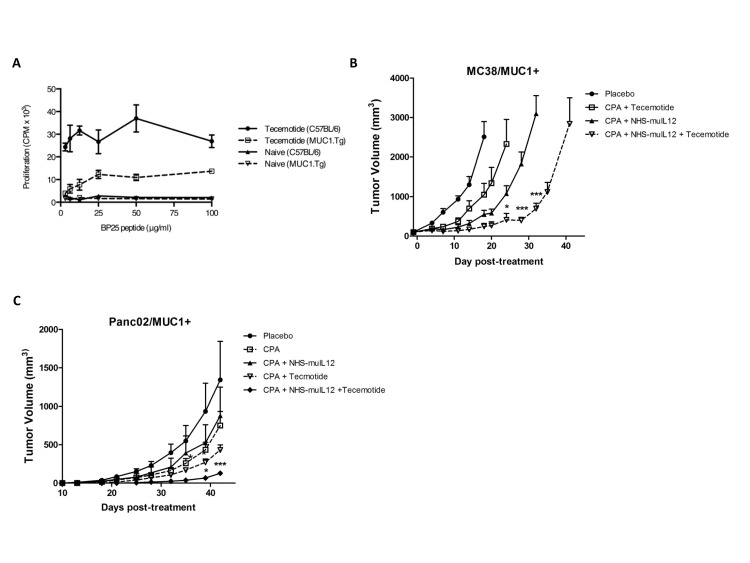
The combination of NHS-muIL12 with an experimental cancer vaccine enhances the anti-tumor response (A) Wild-type C57BL/6 and MUC1.Tg mice were treated with cyclophosphamide and then vaccinated 3 times with tecemotide. Isolated CD4+ splenocytes from vaccinated and naïve mice of each strain were stimulated *in vitro* with MUC1 BP25 peptide, and lymphoproliferation was measured by [^3^H]-thymidine incorporation. Results show mean proliferation (counts per minute) ± SE of triplicate wells for each condition. (B–C) MUC1.Tg mice bearing (B) MC38/MUC1+ s.c. tumors or (C) Panc02/MUC1+ orthotopic tumors were randomized and treated with control liposomes, cyclophosphamide and tecemotide, cyclophosphamide and NHS-muIL12, or the combination of cyclophosphamide, tecemotide, and NHS-muIL12 (*n* = 9-10 mice per group). Cyclophosphamide (2 mg i.v.) was administered on day 0. NHS-muIL12 (10 μg s.c.) was given on day 1. Tecemotide (4x25 μg per dose) was administered s.c. on days 1, 8, 15, and 22. Results show mean tumor volume ± SE over time for each treatment group. Differences in mean tumor volume between groups treated with cyclophosphamide, tecemotide, and NHS-muIL12 were statistically significant versus groups treated with cyclophosphamide and NHS-muIL12 or tecemotide (2-way ANOVA with Bonferonni's post-test; *, *P* < 0.05; ***, *P* <0.001).

NHS-muIL12 treatment was combined with the vaccine tecemotide [27, 28], which contains a 25-mer peptide from the VNTR region of the MUC1 tumor antigen. Tecemotide induced a potent CD4+ T-cell proliferative response to BP25 peptide in C57BL/6 mice and to a lesser degree in C57BL/6 MUC1 transgenic (Tg) mice (Figure 6A). This vaccine was then tested in tumor models together with NHS-muIL12 to determine their combined therapeutic potential. MUC1.Tg mice bearing subcutaneous MC38/MUC1+ tumors were treated with a single injection of cyclophosphamide (100 mg/kg intravenous (i.v.)) 1 day prior to treatment with NHS-muIL12 (10 μg s.c.) and tecemotide (100 μg peptide s.c.). Three weekly booster vaccinations of tecemotide were given following this priming vaccination. Mice treated with the full combination of cyclophosphamide, tecemotide, and NHS-muIL12 showed an increased delay in tumor growth relative to untreated controls and mice that received only 2 therapeutic agents (2-way ANOVA, *P* < 0.001) (Figure 6B). An analogous study in the orthotopic Panc02/MUC1+ tumor model gave similar results (2-way ANOVA, *P* < 0.001) (Fig. 6C).

### Combination therapy with NHS-muIL12 and approved standard of care agents/modalities

**Figure 7:**
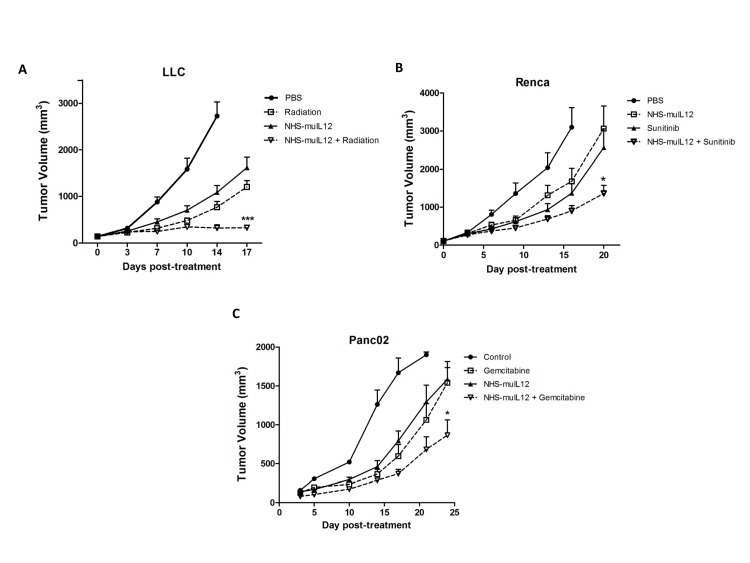
The combination of NHS-muIL12 with fractionated radiotherapy or chemotherapy enhances the anti-tumor response (A) Beginning on day 0, LLC tumor-bearing mice were randomized (*n* = 9 mice per group) and treated with PBS, fractionated radiotherapy alone (360 cGy on days 0-4), NHS-muIL12 alone (10 μg s.c. on day 0), or the combination of fractionated radiotherapy plus NHS-muIL12. (B) Beginning on day 0, Renca tumor-bearing Balb/c mice were randomized (*n* = 10 mice per group) and treated with PBS, sunitinib alone (20 mg/kg orally on days 0-6), NHS-muIL12 alone (10 μg s.c. on day 1), or sunitinib plus NHS-muIL12. (C) Panc02 tumor-bearing mice were randomized (*n* = 11 mice per group) and treated with PBS, gemcitabine alone (120mg/kg i.v. on days 0 and 14), NHS-muIL12 alone (10 μg s.c. on day 0), or gemcitabine plus NHS-muIL12. Results show mean tumor volume ± SE over time for each treatment group. Differences between groups treated in combination were statistically significant versus groups that received monotherapies (2-way ANOVA with Bonferonni's post-test; *, *P* < 0.05; ***, *P* <0.001).

Radiation treatment is known to induce some immune-associated changes in tumor cells, including upregulation of MHC class I molecules and diversification of the intracellular antigenic peptide pool, resulting in better anti-tumor immunity [29]. In mice bearing LLC tumors, the combination of NHS-muIL12 with localized, fractionated radiotherapy showed additive tumor growth inhibition (Figure 7A) that was statistically significant relative to either therapy given alone (2-way ANOVA, *P* < 0.001). The therapeutic combination of radiation with NHS-muIL12 in the MC38 s.c. tumor model showed similar results (data not shown).

Sunitinib is a receptor tyrosine kinase inhibitor used to treat late-stage kidney cancer and gastrointestinal stromal tumor [30] that has been shown to modulate immune responses [31, 32]. The treatment combination of sunitinib with NHS-muIL12 showed an additive improvement in the level of Renca tumor growth inhibition (Figure 7B) that was statistically significant compared to either monotherapy (2-way ANOVA, *P* < 0.001). Gemcitabine is a nucleoside analog used to treat pancreatic cancer and some other carcinomas [33]. Mice bearing orthotopic Panc02 tumors were given 2 treatments of 10 μg NHS-muIL12 (s.c.) plus 120 mg/kg gemcitabine (i.v.) spaced 1 week apart. This treatment combination also showed additive anti-tumor effects (Figure 7C) relative to either monotherapy (2-way ANOVA, *P* < 0.050).

**Figure 8:**
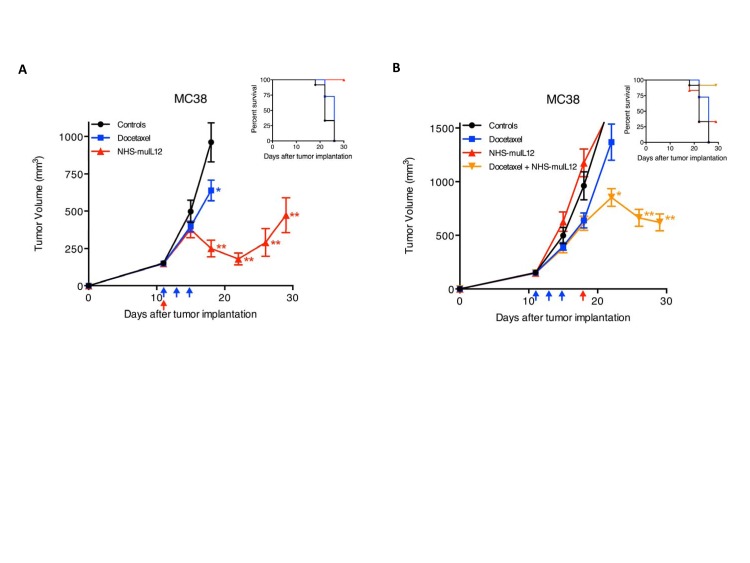
Docetaxel and NHS-muIL12 are effective as monotherapies and combined therapies in mice bearing well-established MC38 tumors (A) Mice bearing well-established (~150 mm^3^) s.c. MC38 tumors were randomized (*n* = 12 mice per group) and treated with saline (closed circle), 3x0.5 mg docetaxel (closed square) on days 11, 13 and 15 (blue arrows), or 1x50 μg NHS-muIL12 (closed triangle) on day 11 (red arrow) after tumor implantation. Docetaxel and NHS-muIL12 each significantly delayed tumor growth as compared with saline-treated controls (1-way ANOVA with Bonferroni's post-test; *, *P* < 0.05; **, *P* < 0.0001). (B) To study the combined efficacy of these therapeutic agents, mice bearing MC38 tumors were randomized (*n* = 12 mice per group) and treated with saline (closed circle), 3x0.5 mg docetaxel (closed square) on days 11, 13 and 15 (blue arrows), 1x50 μg NHS-muIL12 (closed triangle) on day 18 (red arrow), or the sequential combination of both (closed inverted triangle). Graphs show mean tumor volume ± SE over time for each treatment group. The sequential treatment combination resulted in significantly greater anti-tumor effects than saline-treated controls (1-way ANOVA with Tukey's post-test; *, *P* < 0.05; **, *P* < 0.0001). Inserts in both panels A and B indicate percent survival of each treatment group over time following tumor implantation.

Docetaxel is an anti-mitotic taxane that can promote tumor susceptibility to CTL-mediated killing [34]. Mice bearing well-established s.c. MC38 tumors were treated with docetaxel or NHS-muIL12 alone or in combination. Docetaxel treatment of mice bearing ~150mm^3^ MC38 tumors slowed tumor growth, whereas NHS-muIL12 administration resulted in significant transient tumor regression (Figure 8A). The combined administration of docetaxel followed by NHS-muIL-12 was able to induce the sustained regression of very large (~640mm^3^) MC38 tumors (Figure 8B).

## DISCUSSION

Interleukin-12 is clearly capable of eliciting substantial immunotherapeutic effects in various preclinical studies, but its clinical potential has yet to be successfully translated [35-38]. Developing new IL-12 delivery systems to maximize cytokine levels directly in the tumor microenvironment may be a safer and more effective approach to treat cancer. In the present studies, IL-12 has been engineered into an immunocytokine (NHS-IL12) with tumor-targeting capabilities and tested for efficacy. This selective targeting of NHS-IL12 is mediated by the immunoglobulin portion of the molecule (NHS76) that binds to DNA in necrotic tissue [22]. In both mouse and human tumors, rapid tumor growth often outpaces the development of blood vessels, leading to a lack of adequate perfusion and subsequent necrosis. Necrosis can expose intracellular antigens within the tumor microenvironment, which presents an ideal opportunity for the tumor-specific targeting of NHS-IL12. Other IL-12-based immunocytokines have been engineered previously [39,40], but the antibody portion of NHS-IL12 may give it a unique potential to target a wider range of solid tumors.

Here we have shown that NHS76 fused to human IL-12 has a longer half-life *in vivo* but induces lower levels of IFN-gamma release by immune cells both *in vitro* and *in vivo* than IL-12 alone. Furthermore, an NHS76-murine IL-12 conjugate is able to target subcutaneous tumors *in vivo*, and this immunocytokine is superior to an equimolar dose of rMuIL-12 in delaying the growth of 3 different tumor models (LLC, MC38 and B16). The effects of NHS-muIL12 on subcutaneous tumor growth were dose-dependent over a 50-fold range, and NHS-muIL12 showed anti-tumor efficacy after a single injection. The finding that NHS-muIL12 does not directly inhibit MC38 tumor cell growth *in vitro*, combined with the previously reported immune-activating effects of IL-12, provided evidence for the immune-mediated mechanisms by which NHS-muIL12 affects tumor growth *in vivo*.

NHS-muIL12 was found to increase serum IFN-gamma levels, upregulate MHC class I protein expression on DCs, and induce the proliferation of CD49b+ NK cells and CD8+ T cells, all in a dose-dependent manner. An immune cell subset depletion study confirmed that the anti-tumor effects of NHS-muIL12 require CD8+ cells and may involve NK cells, consistent with the known mechanisms of action of IL-12 [41]. Further investigations into the antigen specificity of the CD8+ T-cell response focused on the p15E endogenous retroviral tumor antigen. ELISPOT analysis revealed that mice bearing MC38 or B16 tumors developed a stronger p15E-specific CTL response following NHS-muIL12 treatment. Mice that were cured of their MC38 tumors showed some resistance to tumor rechallenge and displayed long-lasting p15E-specific CTL memory. Thus fusing IL-12 to NHS76 does not alter the fundamental immune-activating mechanisms of the cytokine. The potential of NHS-muIL12 as an effective immunotherapeutic agent is underscored by the significant anti-tumor activity and CTL activation observed following a single s.c. injection.

NHS-muIL12 is clearly more potent against murine tumor models when directly compared with rMuIL-12. This enhanced efficacy of NHS-muIL12 likely stems from the more favorable pharmacokinetic properties of the immunocytokine and its DNA-binding ability at necrotic sites. Additionally, large macromolecules are known to selectively accumulate in tumors through a passive process termed the “enhanced permeabilization and retention effect” [42], which may also apply to immunocytokines. Tumor targeting of NHS-muIL12 may enhance the activation of CD8+ T cells at the site of the tumor and also prolong their cytotoxic effector function. The duration of CD8+ T-cell exposure to IL-12 correlates with the overall level of T-cell activation [43], and it is well known that regulatory T cells (Tregs), macrophages, and other cells found within tumors can be immunosuppressive [44]. The advantage gained by NHS-muIL12 administration is an increase in intratumoral IL-12 concentration, which seems to be biologically important to fully activate tumor antigen-specific CD8+ T cells and overcome this immunosuppressive tumor microenvironment.

In addition to increasing its therapeutic efficacy, the biochemical properties of NHS-IL12 might mitigate the toxicity that has been associated with rHuIL-12. *In vitro* assays using IFN-gamma production to compare functional potency revealed that NHS-huIL12 and NHS-muIL12 were only 5% as potent as molar equivalent amounts of human and murine rIL-12, respectively. Additionally, NHS-muIL12 was extremely well tolerated even at very high doses throughout our studies in mice. Relying on mice alone may not be appropriate for the preclinical modeling of IL-12 toxicity and it is therefore important to note that NHS-IL12 was also well tolerated by monkeys. Injection of cynomolgus monkeys with 40 μg/kg was well tolerated as evidenced by the lack of fever and any change in blood profiles. The immunogenicity against NHS76 seen in mice and monkeys indicates that only clinical trials can provide meaningful information on the repeated administration of NHS-IL12.

Pharmacokinetic data in cynomolgus monkeys and tissue distribution studies in mice indicate that subcutaneous injection is the preferred route of administration of NHS-IL12. The frequency of NHS-IL12 dosing is another important point of consideration, as it has been reported that clinical IL-12-related toxicity is highly schedule-dependent [16]. We have shown here that bolus and fractionated dosing of NHS-muIL12 had equivalent efficacy against MC38 tumors at 2 different total dose levels. This finding indicates that less frequent dosing in clinical studies of NHS-IL12 might reduce toxicity without a loss of efficacy.

One might expect NHS-IL12 to provide additive or synergistic effects when combined with other types of cancer therapy. Many cancer vaccines that were specifically designed to induce strong tumor-specific T-cell responses are in development [45]. However, effector T cells can still be inactivated upon entering the immunosuppressive tumor microenvironment [46]. Pairing a cancer vaccine with NHS-IL12 treatment might allow for the vaccine-induced T cells to better infiltrate the tumor and remain active by virtue of their exposure to NHS-IL12. In 2 preclinical tumor models, the treatment combination of NHS-muIL12 with the vaccine tecemotide was found to delay tumor growth more effectively than either therapy alone. Combining NHS-IL12 with radiation or a chemotherapeutic agent is another attractive option for clinical development. Radiation, docetaxel, sunitinib, and gemcitabine treatment of tumor-bearing mice have all shown favorable immune changes at the tumor site (i.e., antigen modulation, reduced Treg numbers, and support of memory T-cell generation) [29, 34, 47, 48]. In addition to their own anti-tumor mechanisms, these therapies could enhance tumor necrosis and thereby increase the tumor-targeting capacity of NHS-IL12. Pairing NHS-muIL12 with radiation, sunitinib, gemcitabine, and docetaxel in the preclinical studies reported here showed additive anti-tumor effects, indicating that each of these treatment combinations might be worth investigating in the clinic.

A phase I clinical trial of NHS-IL12 in patients with metastatic or locally advanced solid tumors has recently been initiated to determine the maximum tolerated dose (MTD) and optimal biological dose (clinical trial #NCT01417546). Once the MTD of NHS-IL12 is identified, the studies reported here will provide the rationale for phase II trials that combine NHS-IL12 with other experimental or standard of care cancer therapies.

## MATERIALS AND METHODS

### Ethics statement

Investigation has been conducted in accordance with the ethical standards and according to the Declaration of Helsinki and according to national and international guidelines and has been approved by the authors' institutional review board.

### Animal models and tumor cell lines

Adult female C57BL/6, Balb/c, and athymic mice were purchased from NCI-Frederick and Charles River Laboratories. Mice that express the human CEA gene on the C57BL/6 background were kindly provided by Dr. John Shively (City of Hope, Duarte, CA). C57BL/6 mice expressing the human MUC1 gene were a gift from Dr. Sandra Gendler (Mayo Clinic, Scottsdale, AZ) and used for tecemotide studies. All animals were housed and maintained under pathogen-free conditions in microisolator cages, and were 2–6 months old at the start of each study. Animal care was in compliance with the recommendations of *The Guide for Care and Use of Laboratory Animals* (National Research Council).

Lewis lung carcinoma (LLC), B16, MC38, and Renca cells were cultured in DMEM containing 10% heat-inactivated fetal bovine serum. CEA-expressing and MUC1-expressing MC38 cells were cultured in the same media supplemented with 300 μg/ml G418. At the start of each study, tumor cells were trypsinized, washed, resuspended in HBSS, and then injected s.c. into the right rear flank of each mouse. Mice were anesthetized with ketamine (15 mg/kg) and xylazine (75 mg/kg) and shaved on the rear flank prior to subcutaneous tumor cell injection.

### Treatments

NHS-huIL12 consists of the NHS76 DNA-binding antibody [22] fused to 2 molecules of the human IL-12 heterodimer. NHS-muIL12 consists of the same human NHS76 antibody fused to 2 molecules of the murine IL-12 heterodimer. NHS-huIL12 was produced by transient transfection of CHO-S cells, and NHS-muIL12 and the control immunocytokine BC1-muIL12 were produced by transient transfection of HEK293. All three immunocytokines were purified by Protein A chromatography. The antigen-binding and IL-12 stimulatory functions of the fusion proteins were characterized *in vitro* (data not shown) prior to the conduct of *in vivo* studies. For anti-tumor efficacy studies, NHS-muIL12 or rMuIL-12 (R&D Systems) was injected s.c. into the inner leg contralateral to the tumor.

Tecemotide is a liposomal vaccine encapsulating monophosphoryl lipid A and a 25-mer peptide derived from the human MUC1 tumor antigen [27, 28]. Lyophilized tecemotide was obtained from Merck KGaA and resuspended in PBS prior to injection at a concentration of 0.5 mg peptide/ml. To help overcome immune tolerance to MUC1, mice were first injected once i.v. with saline containing 2 mg cyclophosphamide (Sigma) into the tail vein 1-3 days prior to the first tecemotide vaccination. Mice were then shaved in the abdominal area and injected s.c. with a weekly dose of tecemotide containing 25 μg peptide at each of 4 sites (100 μg total peptide per dose).

Sunitinib powder was obtained as a free base from Merck KGaA and dissolved to a working concentration of 2 mg/ml. The pH was adjusted to 6.0 and the drug was administered to mice orally at 20 mg/kg/day for 7 consecutive days.

Gemcitabine hydrochloride was obtained from Eli Lilly, diluted in 0.9% saline to a concentration of 12mg/ml, and injected i.v. into the tail vein at a dose of 120mg/kg. Mice received 2 separate injections spaced 14 days apart.

Docetaxel solution was obtained from Hospira, diluted in 0.9% saline to a concentration of 5mg/ml, and injected i.p. at a dose of 0.5mg/mouse. Mice received 3 separate injections spaced 2 days apart.

### *In vitro* bioactivity assays

Freshly isolated normal human PBMCs were incubated with phytohemagglutinin (2 μg/ml, PHA-P) for 4 days to promote the formation of blastoid cells. Human IL-2 (R&D Systems) was then added for an additional day, in order to upregulate the expression of the IL-12 receptor on these cells. NHS-huIL12 and rHuIL-12 (R&D Systems) were then added and supernatants were harvested 24 h later. Secreted IFN-gamma levels were measured using an enzyme-linked immunosorbent assay (ELISA).

### Cynomolgus monkey studies

Cynomolgus macaques were treated with 40 μg/kg NHS-huIL12 s.c., 40 μg/kg NHS-huIL12 i.v., or 4 μg/kg rHuIL-12 i.v. (*n* = 2/group). Serum levels of IFN-gamma were monitored for 5 days post-injection by ELISA. Drug levels were monitored for 8 days post-injection by IL-12 ELISA.

### Noninvasive *in vivo* imaging

NHS-muIL12 and BC1-muIL12 were conjugated to Alexa Fluor 750 using the SAIVI Rapid Antibody Labeling Kit (Invitrogen) and injected either s.c. or i.v. into nude mice bearing Lewis lung tumors. Fluorescence and photographic images of the tumor site were taken over the course of 3 days using an IVIS 200 imaging system (Caliper Life Sciences). Fluorescence at the site of the tumor was analyzed using Living Image software (Caliper Life Sciences).

### Immunohistochemical detection

Formalin-fixed, paraffin-embedded LLC tumors were sectioned at 3 μm and mounted onto Super Frost slides. Following deparaffinization, the slides were placed in an automated staining instrument (Ventana Medical Systems). Slides were incubated with a primary Ab (rabbit anti-huIgG1; Epitomics) for 8 h at room temperature followed by incubation with a secondary Ab (anti-rabbit IgG-HRP; OmniMap) for 16 minutes at 37˚C. Chromogenic detection was made using ChromoMap DAB substrate solution (Ventana). Counterstaining was performed with Hematoxylin II (Ventana) and a post-counterstain was performed using Bluing Reagent (Ventana).

### Subcutaneous tumor growth studies

Mice were inoculated with LLC (5×10^5^), B16 (5×10^5^), MC38 (3−5×10^5^), MC38/CEA+ (3×10^5^), MC38/MUC1+ (10^6^), or Renca (10^6^) tumor cells in the right rear flank. Tumors became measurable 7-12 days later at which time the mice were randomly assigned to experimental groups and treatment was initiated. Tumors were measured twice per week throughout the studies using calipers, and the tumor volume was calculated as: Volume = (width)^2^ × (length) × 0.5236. Mice were euthanized when the tumor volume exceeded 1.5cm^3^.

### Orthotopic pancreatic tumor growth studies

Previously frozen Panc02 or Panc02/MUC1+ tumor fragments were surgically implanted onto the pancreases of female C57BL/6 or MUC1.Tg mice. Twelve days later, ~2mm^3^ tumor fragments were prepared from these seed tumors and implanted onto the pancreas of each mouse on the study. Treatment was initiated 7 days after tumor implant. To monitor tumor growth, mice were anesthetized and tumor diameter was measured with calipers. Tumor volume was calculated as: Volume = (4/3) × 3.14159 × (Diameter/2)^3^. Mice were euthanized when the tumor volume exceeded 2.0cm^3^.

### Radiotherapy combination study

Mice were inoculated into the right quadriceps muscle with 5×10^5^ LLC cells. Ten days after inoculation, mice were sorted into treatment groups with mean tumor volumes of ~135mm^3^. Tumor-bearing legs were irradiated (360 cGy daily/5 days) by timed exposure to a ^137^Cs source (GammaCell 40 Exactor); a lead collimator device was used to localize delivery. Tumors were measured twice per week throughout the study using calipers, and the tumor volume was calculated as: Volume = (length) × (width) × (height)/2. Mice were euthanized when the tumor volume exceeded 2.0cm^3^.

### Serum cytokine quantification

Mouse serum was obtained 48 h following s.c. NHS-muIL12 treatment of non-tumor-bearing C57BL/6 mice. Murine IFN-gamma levels were determined using a standard ELISA kit (Thermo Fisher Scientific) according to the manufacturer's instructions. Sample ODs at 450nm were read using a Synergy HT plate reader (Bio-Tek).

### MHC expression by dendritic cells

Single-cell suspensions prepared from spleens were labeled with an antibody cocktail containing anti-mouse-CD11c-PE-Cy7, anti-mouse-CD80-APC, anti-mouse-CD86-PE, anti-mouse-H-2K^b^-FITC, and anti-mouse-I-A^b^-Pacific Blue (all antibodies were purchased from eBioscience). The mean fluorescence intensities (MFI) of H-2K^b^ (MHC class I) and I-A^b^ (MHC class II) in the CD11c^+^CD80^high^CD86^high^ population were determined using a BD LSR II flow cytometer and BD FACSDiva software.

### *In vivo* lymphocyte proliferation

Mice were administered 1mg of bromodeoxyuridine (BrdU) via intraperitoneal (i.p.) injection at 24 h, 16 h, and 1 h prior to harvest of tumors and spleens. Minced tumor slurries were dissociated in media containing collagenase IV (400 units/ml; Worthington) and DNase I (100 μg/ml; Roche). Single-cell suspensions were prepared from spleens and labeled with an antibody cocktail containing anti-mouse CD45-PerCP-Cy5.5, anti-mouse CD8-Pacific Blue, anti-mouse CD4-Alexa Fluor 750, and anti-mouse CD49b-APC (eBioscience). Intracellular antibody staining against BrdU was performed using a commercially available kit (BD) according to the manufacturer's instructions. Data were acquired on a BD LSR II flow cytometer and analyzed using FACS Diva software.

### Immune cell subset depletions

To deplete CD4+ or CD8+ T-cell populations, mice were given 4 daily i.p. injections of 100 μg GK1.5 or 2.43 antibodies, respectively, the week before tumor implantation. Mice were then given additional weekly i.p. injections throughout the study to maintain these low T-cell levels. NK cells were depleted by injecting mice once per week with 25μl rabbit anti-asialo-GM1 (Cedarlane Laboratories), beginning the week before tumor cell injection and continuing through the end of the study.

### Anti-drug antibody detection in mice

C57BL/6 mice (*n* = 4/group) received 2 s.c. injections of NHS-muIL12 (10 μg) or PBS given 7 days apart. Plasma was collected 1 week after the last administration. Antibodies against NHS-muIL12 were detected using an ELISA method: 96-well immunoassay plates (Nunc) were coated by incubation with 2 μg/ml NHS-muIL12 in carbonate buffer overnight, followed by blocking with 5% BSA. Plasma samples were serially diluted in the assay plate and incubated for 1 h. Detection was made using a rabbit anti-mouse IgG1 antibody conjugated to horse-radish peroxidase (Jackson ImmunoResearch) and TMB substrate (BioFX). A standard curve ranging from 1.56 to 100ng/ml was prepared using mouse IgG1 (Invitrogen). Sample ODs at 450 nm were read using a SpectraMax 340PC plate reader (Molecular Devices).

### ELISPOT assay

Pooled splenocytes from each study group were stimulated *in vitro* with 1 μg/ml of p15E peptide (KSPWFTTL, H-2K^b^). Six days later, CD8+ T cells were isolated by magnetic-bead separation (Miltenyi) and analyzed using an IFN-gamma ELISPOT kit (BD Biosciences) according to the manufacturer's instructions. A total of 20,000 CD8+ T cells were co-incubated with splenocytes pulsed with either p15E peptide or a negative control OVA peptide (SIINFEKL, H-2K^b^). As positive controls, CD8+ T cells were stimulated with 1 μmol/L of phorbol 12-myristate 13-acetate and 10 ng/mL of ionomycin. Co-cultures were carried out in triplicate. Spot counts were analyzed using an ELISPOT plate reader (Immunospot).

### CTL assay

A CD8+ T-cell functional assay was carried out using RPMI 1640 containing 10% heat-inactivated fetal bovine serum, 2 mM L-glutamine, 50 μM β-mercaptoethanol, 0.1 mM nonessential amino acids, 15 mM Hepes buffer (pH 7.4), 1 mM sodium pyruvate, and penicillin/streptomycin. Splenocytes from mice that became tumor-free after NHS-muIL12 treatment were stimulated *in vitro* with 10 μg/ml p15E peptide (KSPWFTTL, H-2K^b^). After 6 days, these cells were co-incubated in triplicate wells of round-bottom 96-well plates with ^111^In-labeled EL-4 cells pulsed with 10 μg/ml p15E peptide or a negative control VSV-NP peptide (RGYVYQGL, H-2K^b^). After overnight incubation, ^111^In release was measured via gamma counter and cytotoxicity was calculated as % specific lysis = [(experimental cpm - spontaneous cpm) / (total cpm – spontaneous cpm)] x 100. Results from triplicate wells were combined and reported as mean ± SE.

### *In vitro* MUC1-specific CD4+ lymphocyte proliferation assay

The 25-mer peptide used to produce tecemotide (BP25, STAPPAHGVTSAPDTRPAPGSTAPP) was synthesized and purified by CPC Scientific. Two weeks after the final tecemotide booster vaccination, spleens from naïve and vaccinated mice were harvested and prepared as single cell suspensions. CD4+ splenocytes were isolated using a Dynal CD4 cell negative isolation kit (Invitrogen) according to the manufacturer's instructions. These cells (2×10^5^/well) were then co-incubated with irradiated splenocytes (20 Gy, 10^6^/well) from syngeneic naïve mice and 3.1-100 μg/ml of BP25 peptide in flat-bottom 96-well plates. After 4 days, [^3^H]-thymidine (1μCi/well) was added to each well and incubated overnight. The cells were harvested onto glass fiber filtermats using a Tomtec Harvester 96, and ^3^H incorporation was measured by liquid scintillation counting on a 1450 MicroBeta counter (Perkin-Elmer). Each sample was run in triplicate, and results were combined to yield a mean ± SE.

### Statistical analysis

For subcutaneous and orthotopic tumor growth studies, the tumor volume over time was plotted as mean ± SE. Survival comparisons between treatment groups were made using Kaplan-Meier plots and analyzed with the log-rank test. To analyze the differences in mean tumor volume between treatment groups, the latest time point at which all mice remained alive was selected for 1-way or 2-way ANOVA analysis followed by an appropriate post-test. The student's *t*-test was used to analyze a study with only 2 treatment groups. Analyses were carried out using the GraphPad Prism software package (Prism 5 for Mac OS X, version 5.0d, GraphPad Software, Inc.). Statistical significance was accepted if *P* < 0.05.

## SUPPLEMENTARY FIGURE


