# Supplementary material for: The immunocytokine NHS-IL12 as a potential cancer therapeutic
Source: Oncotarget. 2014 Mar 24;5(7):1869–84. doi: 10.18632/oncotarget.1853 (PMC4039112; doi:10.18632/oncotarget.1853)
Supplement: Supplementary file 1 [file oncotarget-05-1869-s001.pdf]

The Immunocytokine NHS-IL12 as a Potential Cancer Therapeutic

SUPPLEMENTAL FIGURES

Supplemental Figure S1

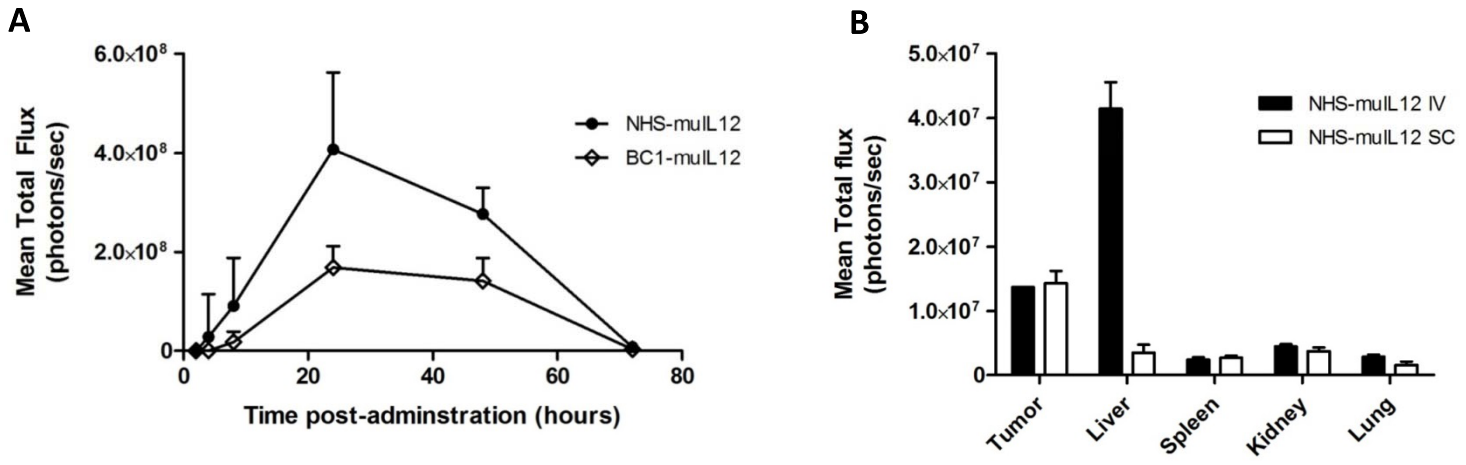

Supplemental Figure S1. NHS-muIL12 targets to LLC tumors following subcutaneous or intravenous administration. Nude mice bearing 300-400mm^3 subcutaneous (s.c.) Lewis lung carcinoma (LLC) tumors were injected with 100 µg of NHS-muIL12 or BC1-muIL12 conjugated to Alexa Fluor 750. *In vivo* fluorescence imaging was used to monitor drug distribution for 3 days following treatment. **(A)** Comparison of tumor localization over time of NHS-muIL12 and BC1-muIL12 following s.c. delivery of each fluorescent immunocytokine. Each point is the mean fluorescence ± SE of 3 mice per group. **(B)** Drug distribution of NHS-muIL12 in tumors and normal tissues at 48 hours following s.c. or i.v. administration. The mean fluorescence ± SE of 3 mice per group is shown.

Supplemental Figure S2

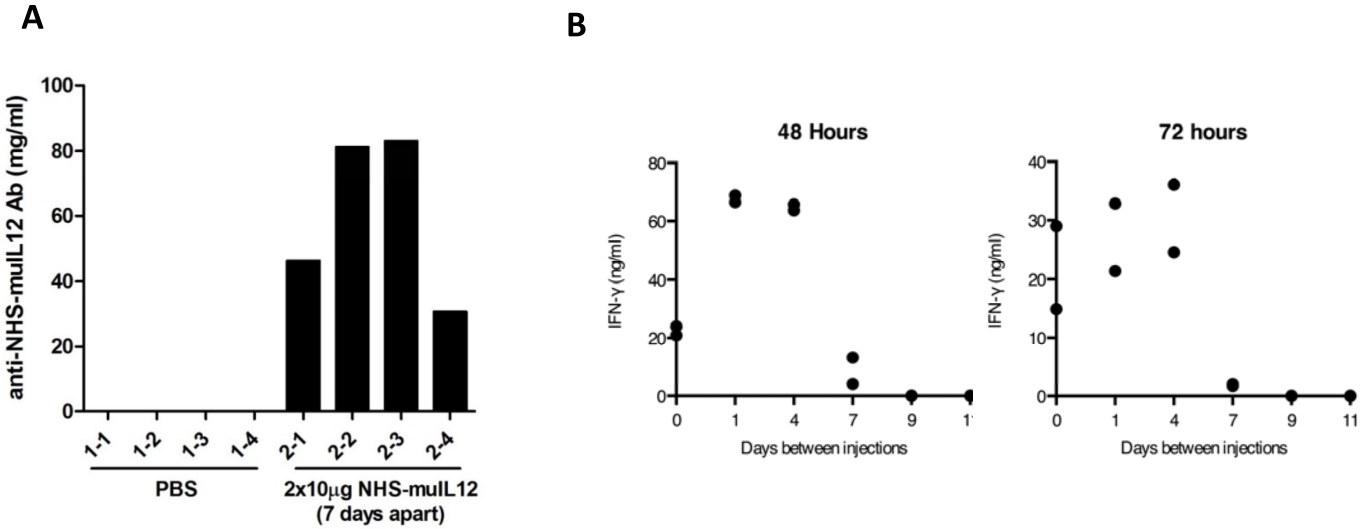

Supplemental Figure S2. NHS-muIL12 is highly immunogenic in C57BL/6 mice. **(A)** Anti-NHS-muIL12 antibody responses in 4 control C57BL/6 mice and 4 mice that were treated with 2 s.c. doses of 10 μg NHS-muIL12 spaced 7 days apart. **(B)** Serum IFN-γ levels at 48 hours and 72 hours after a second injection of 5 μg NHS-muIL12 that was given 1, 4, 7, 9, or 11 days after the initial injection.

Supplemental Figure S3

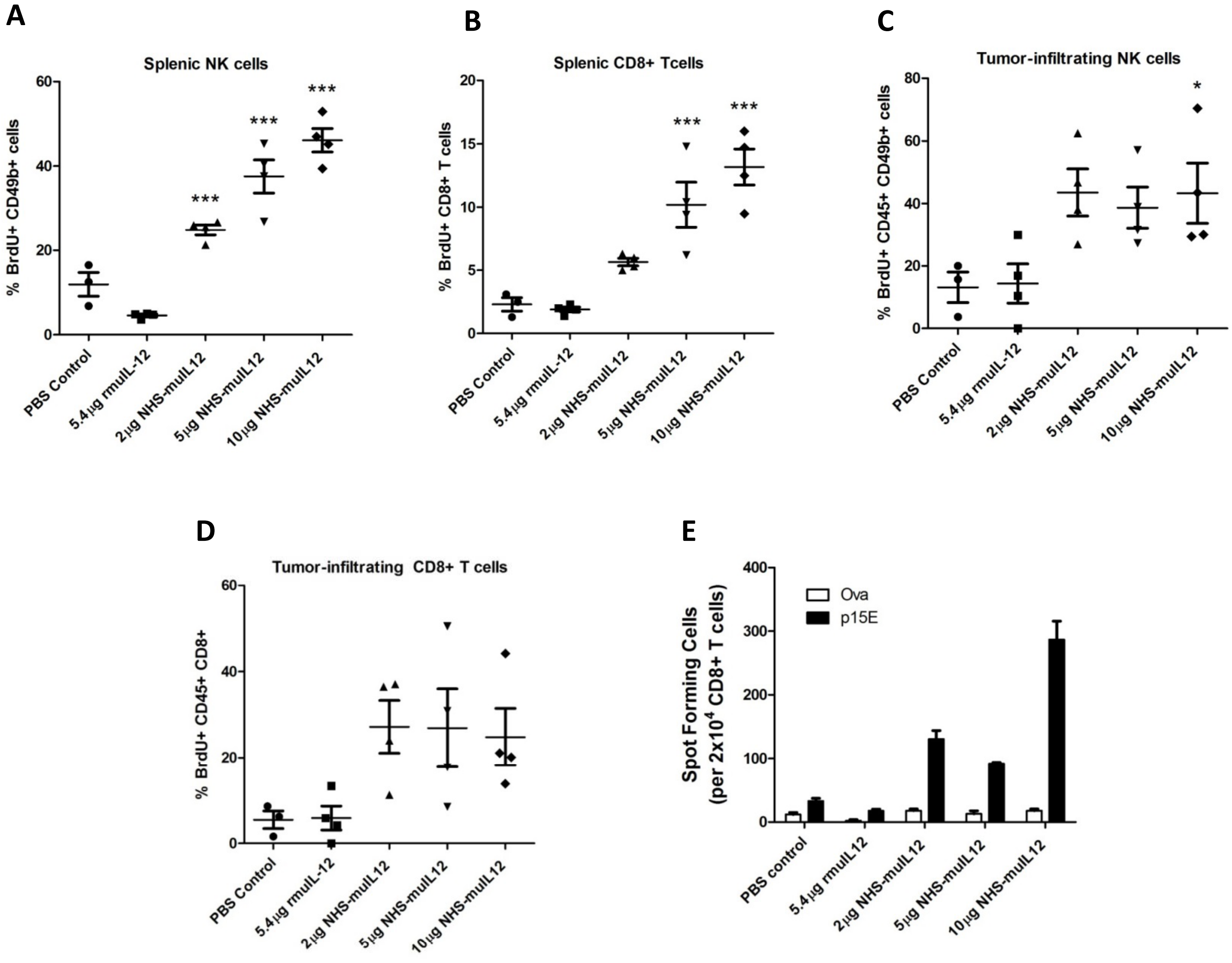

Supplemental Figure S3. NHS-muIL12–mediated activation of cellular immunity is dose-dependent in B16 tumor-bearing mice. B16 tumor-bearing mice were randomized and treated with PBS (●), 5.4 μg rMuIL-12 (■), 2 μg NHS-muIL12 (▲), 5 μg NHS-muIL12 (▼), or 10 μg NHS-muIL12 (◆). Five days after treatment, flow cytometry was used to quantify BrdU incorporation by **(A)** splenic CD49b+ NK cells, **(B)** splenic CD8+ T cells, **(C)** tumor-infiltrating CD49b+ NK cells, and **(D)** tumor-infiltrating CD8+ T cells in treated mice. Asterisks indicate statistically significant differences between NHS-muIL12 treated groups versus control and rmuIL-12 treated groups (1-way ANOVA with Bonferonni's post-test; *, P <0.05; ***, *P* <0.001.) **(E)** ELISPOT assays were used to determine the frequency of splenic CD8+ T cells specific for p15E or ovalbumin after pooling the splenocytes from *n* = 4 mice/group; error bars represent SEM of triplicates.
